# Nitric oxide attenuates PI4P accumulation at the ER membrane to inhibit encephalomyocarditis virus replication selectively in β-cells

**DOI:** 10.1016/j.jbc.2025.110798

**Published:** 2025-10-09

**Authors:** Alyssa L. Gehant, Joshua D. Stafford, Polly A. Hansen, Katherine R. Harty, Aaron Naatz, John A. Corbett

**Affiliations:** Department of Biochemistry, Medical College of Wisconsin, Milwaukee, Wisconsin, USA

**Keywords:** type 1 diabetes, T1D, β-cell, nitric oxide, picornavirus, EMCV, PI4K, PI4KIIIα, PI4P

## Abstract

Viral infection, particularly by members of the picornavirus family, has been associated with autoimmune diabetes (type 1 diabetes mellitus) onset. The encephalomyocarditis virus (EMCV) is a mouse-tropic member of the picornavirus family that stimulates innate immune activation, leading to the production of cytokines. In response to cytokines, β-cells express inducible nitric oxide (NO) synthase and produce low micromolar levels of the free radical, NO. We have previously shown that, because of its inhibitory action on mitochondrial oxidation and depletion of cellular ATP, NO selectively attenuates EMCV replication in, and lysis of, β-cells. In this study, we show that one mechanism by which NO inhibits EMCV replication is by attenuating the accumulation of phosphatidylinositol-4-phosphate at the endoplasmic reticulum membrane. As a result, viral replication complex formation is prohibited, and viral replication is effectively prevented. In agreement with previous studies, we show that these observations are selective for β-cells and because of a loss of cellular ATP.

β-cells reside in pancreatic islets of Langerhans and are responsible for insulin secretion. Type 1 diabetes mellitus (T1D) is defined by the autoimmune destruction of β-cells, resulting in dysregulation of whole-body glucose homeostasis (1, 2). While factors associated with T1D onset remain largely unknown, it is believed that both genetic and environmental factors play a role (3, 4, 5, 6). Genetic predispositions associated with T1D have been studied (3, 4); however, no genetic predisposition has been associated with complete disease penetrance. In addition, the low concordance rate among monozygotic twins supports a role for environmental factors in disease pathogenesis (3, 4).

Viral infection, particularly by members of the picornavirus family, is an environmental factor that has long been associated with T1D development (5, 7, 8, 9). Enteroviruses (such as coxsackie-B virus and poliovirus), which are members of the picornavirus family, hold the strongest association with T1D incidence in humans (10, 11). A review of T1D prevalence studies revealed that the rate of enteroviral infection is 10-fold higher in children with recent T1D diagnosis, when compared with healthy controls (12). In addition, in a longitudinal study of genetically predisposed children expressing islet autoantibodies, individuals who progressed to T1D development progressed more rapidly when seropositive for enteroviral RNA (13). These studies suggest that viral infection by picornavirus family members may participate in T1D onset and/or progression, though a direct causal relationship has yet to be established. Therefore, it is believed that T1D onset requires both a genetic predisposition and an environmental trigger (such as viral infection) for complete disease penetrance (3, 4, 5, 7, 10).

The encephalomyocarditis virus (EMCV) is a mouse-tropic member of the picornavirus family (14). It is a highly inflammatory virus that has been shown to induce diabetes in certain mouse strains, making it an ideal model for mechanistic studies of virus-induced autoimmune diabetes (14). Upon EMCV infection, like enteroviral infection, immune cells rapidly respond with the production of pro-inflammatory cytokines (15, 16, 17). In response to cytokines, β-cells activate inflammatory signaling cascades, resulting in the expression of inducible nitric oxide (NO) synthase (iNOS) and the production of up to micromolar levels of NO (18, 19, 20). Multiple laboratories, including our own, have shown that NO mediates the inhibitory effects of cytokines on insulin secretion in isolated islets and primary rodent β-cells (18, 19, 20, 21, 22). Although seemingly detrimental, we hypothesize that there is a physiological benefit for the endogenous production of NO by β-cells, particularly during a viral infection. In support of this hypothesis, we have shown that, in a β-cell–selective manner, iNOS-derived levels of NO attenuate EMCV replication and EMCV-mediated β-cell lysis (23). NO is an effective inhibitor of mitochondrial oxidative metabolism, targeting aconitase (tricarboxylic acid [TCA] cycle) and complex IV of the electron transport chain (19, 24, 25). Under conditions of mitochondrial inhibition, most cell types generate ATP using anaerobic glycolysis; however, β-cells lack this metabolic flexibility and are unable to replenish NAD^+^ needed for continued glycolysis (26, 27, 28). This is due, in part, to low expression levels of lactate dehydrogenase, and results in a decrease in cellular ATP (19, 20, 26). We have shown that the cell type–selective protection of β-cells against EMCV-mediated lysis is due to the inhibition of oxidative mitochondrial metabolism by NO and the resulting depletion of cellular ATP (23, 29).

In this study, we define a novel mechanism by which NO-mediated ATP depletion in β-cells leads to the inhibition of EMCV replication and EMCV-mediated lysis. We show that NO targets a specific step in the EMCV viral life cycle that is required for efficient replication. Phosphatidylinositol-4-kinase-IIIα (PI4KIIIα) is responsible for the production of phosphatidylinositol-4-phosphate (PI4P) (30, 31, 32, 33). During infection, the host factor function of PI4KIIIα is hijacked, and PI4P production is directed predominantly to the endoplasmic reticulum (ER) membrane (34, 35). Accumulation of PI4P aids in localized cholesterol enrichment, stabilizes viral replication complexes, and allows for efficient viral replication to occur (33, 35, 36, 37). We show that NO attenuates the accumulation of PI4P, causing disruption in replication complex stabilization, and as a result, inhibits EMCV replication.

## Results

### NO attenuates EMCV mRNA and protein accumulation in MIN6 cells

**Figure 1:**
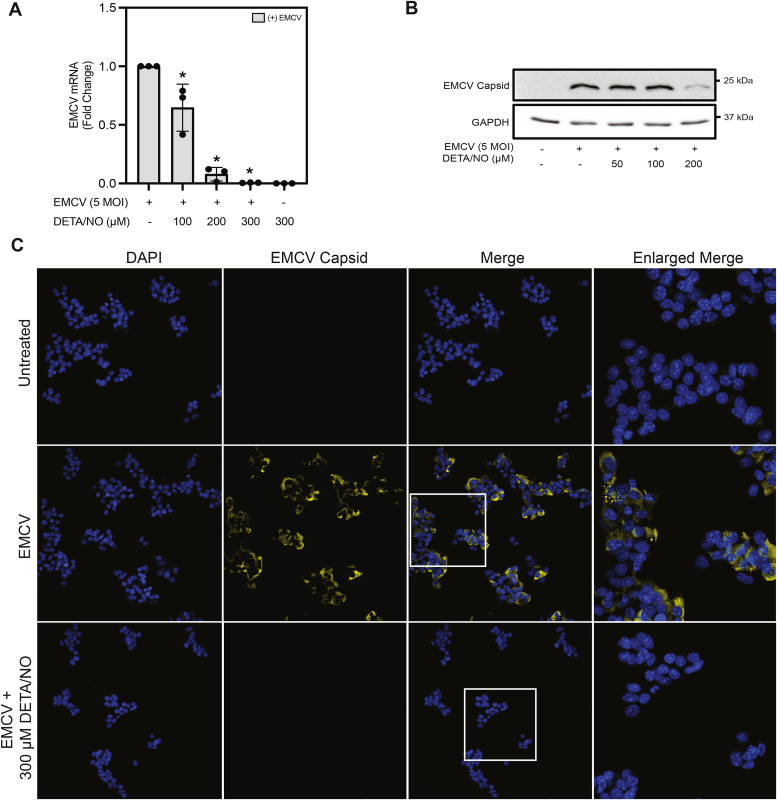
**Nitric oxide attenuates EMCV mRNA and protein accumulation in MIN6 cells.** MIN6 cells (300,000 cells/400 μl) were infected with 5 MOI EMCV with or without indicated concentrations of DETA/NO and EMCV (viral protein-1 [VP1]) mRNA accumulation was measured by RT–qPCR 6 h postinfection (*A*) and EMCV capsid protein by Western blot analysis 18 h postinfection (*B*). MIN6 cells (600,000 cells/800 μl) were infected with 5 MOI EMCV with or without 300 μM DETA/NO for 18 h. Nuclei were identified using DAPI fluorescence (*blue*), and cells containing EMCV were identified by immunostaining for EMCV capsid protein (*yellow*). Cells were visualized at 40× magnification (*C*). Results are the average ± SD of three independent experiments (*A*) or representative of three independent experiments (*B* and *C*). Statistically significant differences are indicated (∗*p* < 0.05). DAPI, 4′,6-diamidino-2-phenylindole; DETA/NO, (Z)-1-(*N*-(2-aminoethyl)-*N*-(2-ammonioethyl)amino)diazen-1-ium-1,2-diolate; EMCV, encephalomyocarditis virus; MOI, multiplicity of infection.

Because EMCV is mouse tropic, and mouse insulinoma cell lines do not respond normally to cytokines with the expression of iNOS, NO donors have been used to study the actions of this free radical on EMCV replication. Consistent with our previous studies (23, 29), treatment of mouse insulinoma (MIN6) cells with the long half-life (∼20 h) NO donor, (Z)-1-(*N*-(2-aminoethyl)-*N*-(2-ammonioethyl)amino)diazen-1-ium-1,2-diolate (DETA/NO), results in a concentration-dependent inhibition of EMCV viral protein-1 (VP1) mRNA accumulation (Fig. 1*A*). Consistent with the inhibition of EMCV mRNA accumulation, EMCV capsid protein is also attenuated by DETA/NO in a similar concentration-dependent manner, as shown by Western blot analysis (Fig. 1*B*) and immunofluorescence staining in MIN6 cells 12 h post-EMCV infection (Fig. 1*C*). These findings are consistent with previous studies showing that NO inhibits the accumulation of EMCV mRNA and β-cell lysis by a mechanism dependent on the inhibition of oxidative mitochondrial metabolism and depletion of cellular ATP (23, 29).

### NO selectively inhibits PI3K-mediated Akt phosphorylation in MIN6 cells

**Figure 2:**
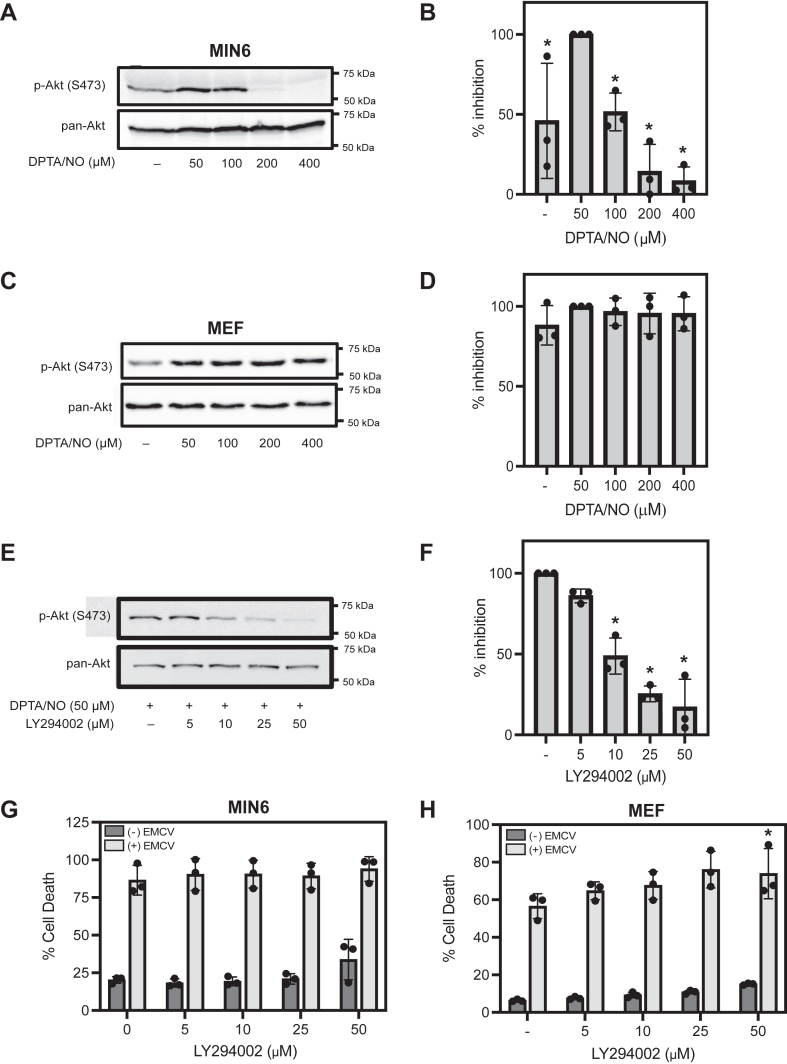
**Effects of nitric oxide on PI3K activity and cell viability in response to EMCV infection.** MIN6 cells (300,000 cells/400 μl) (*A*) and MEF (40,000 cells/400 μl) (*C*) were treated with or without indicated concentrations of DPTA/NO for 1 h, and then Akt phosphorylation (S473) was measured by Western blot. Corresponding densitometric analysis for MIN6 (*B*) and MEF (*D*) are shown. MIN6 cells (300,000 cells/400 μL) treated with or without 50 μM DPTA/NO and indicated concentrations of LY294002 for 1 h, cells were harvested, and Akt phosphorylation (S473) was measured *via* Western blot (*E*), and the corresponding densitometric analysis is shown (*F*). MIN6 cell (75,000 cells/100 μl) (*G*) and MEF cell (10,000 cells/100 μl) (*H*) viability was measured by SYTOX fluorescence 24 h (MIN6 cells) or 18 h (MEF) post-EMCV infection in the presence or the absence of indicated concentrations of LY294002. Results are representative of three independent experiments (*A*, *C*, and *E*) or the average ± SD of three independent experiments (*B*, *D*, *F*, *G*, and *H*). Statistically significant differences are indicated (∗*p* < 0.05). No statistically significant differences were observed in *D*, *G*, and *H*. DPTA/NO, (Z)-1-[*N*-(3-aminopropyl)-*N*-(3-ammoniopropyl)amino]diazen-1-ium-1,2-diolate; EMCV, encephalomyocarditis virus; MEF, mouse embryonic fibroblast.

To understand the molecular mechanism by which NO selectively attenuates EMCV-mediated lysis of β-cells, we made the surprising observation that PI3K signaling, in response to NO, differed in β-cells when compared to non-β-cells. In response to concentrations of (Z)-1-[*N*-(3-aminopropyl)-*N*-(3-ammoniopropyl)amino]diazen-1-ium-1,2-diolate (DPTA/NO) (short half-life NO donor) below 200 μM, NO stimulates the phosphorylation of the PI3K substrate, Akt (at S473), in MIN6 cells (Fig. 2, *A* and *B*). At concentrations of 200 μM or greater, NO fails to stimulate Akt phosphorylation, and in fact, it attenuates Akt phosphorylation to levels below those observed under basal conditions (Fig. 2, *A* and *B*). Like MIN6 cells, DPTA/NO at 50 to 100 μM stimulates PI3K-dependent Akt phosphorylation in mouse embryonic fibroblasts (MEFs); however, at concentrations that inhibit Akt phosphorylation in MIN6 cells (>200 μM), DPTA/NO does not inhibit PI3K activity in MEF (Fig. 2, *C* and *D*).

Even though PI3K signaling is modified by NO, this kinase does not contribute to EMCV-mediated lysis of β-cells. As expected, PI3K inhibition using the PI3K inhibitor, LY294002, attenuates Akt phosphorylation in a concentration-dependent manner in MIN6 cells (Fig. 2, *E* and *F*); however, it does not prevent EMCV-mediated lysis in MIN6 cells or MEF (Fig. 2, *G* and *H*, respectively). These results suggest that differential PI3K activity in response to increasing concentrations of NO is not responsible for the attenuation of EMCV-mediated cell death in β-cells.

### PI4K inhibition results in a cell type–independent attenuation of EMCV-mediated lysis

**Figure 3:**
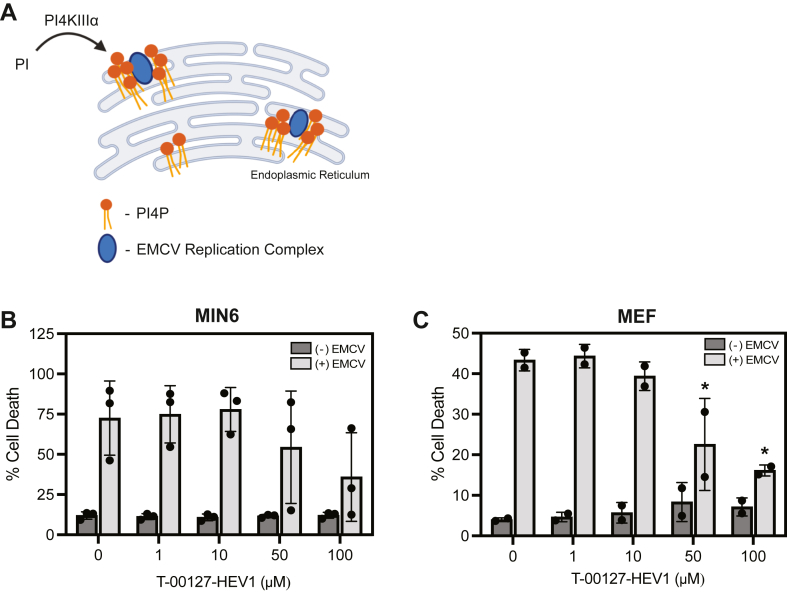
**The effect of PI4K inhibition on EMCV-mediated cell lysis.** Schematic summarizing the role of PI4K in EMCV replication complex formation (*A*). The effects of the PI4K inhibitor, T-00127-HEV1, on MIN6 cell (75,000 cells/100 μl) (*B*) and MEF cell (10,000 cells/100 μl) (*C*) viability following infection with 5 MOI and 0.1 MOI EMCV, respectively. Cell death was measured by SYTOX fluorescence 18 h (MEF) or 24 h (MIN6) postinfection. Results are the average ± SD (*B* and *C*) of three independent experiments. Statistically significant differences are indicated (∗*p* < 0.05). EMCV, encephalomyocarditis virus; MEF, mouse embryonic fibroblast; MOI, multiplicity of infection.

While inhibition of PI3K did not affect EMCV-mediated lysis in MIN6 cells or MEFs, other members of the PI3/4K family of kinases are required for picornavirus replication. More specifically, PI4KIIIβ is known to participate in enteroviral replication (38), whereas PI4KIIIα is believed to be an essential host factor required for EMCV replication (35). Both PI4KIIIα and PI4KIIIβ catalyze the reaction of phosphatidylinositol to PI4P (31). In a healthy cell, PI4P is a highly abundant phospholipid that participates in membrane trafficking, membrane stabilization, ion channel regulation, and vesicle formation (31). During EMCV infection, the host factor function of PI4KIIIα is hijacked, and PI4P accumulates at the ER membrane (35). PI4P accumulation at the ER is required for viral replication complex stabilization, recruitment of other host factors (such as oxysterol-binding protein, OSBP), cholesterol enrichment, and ultimately, efficient EMCV replication (Fig. 3*A*) (34, 35). Since NO, at concentrations that inhibit EMCV-mediated lysis, also attenuates PI3K signaling, we tested the hypothesis that NO similarly attenuates PI4KIIIα activity, resulting in the attenuation of PI4P accumulation and disruption of EMCV replication complex stabilization. To test this hypothesis, the effect of the PI4K inhibitor, T-00127-HEV1, on MIN6 cell viability was examined following EMCV infection (Fig. 3*B*). While T-00127-HEV1 has selectivity for PI4KIIIβ (IC_50_ = 150 nM), it is also an effective inhibitor for PI4KIIIα (IC_50_ = 75 μM) (39, 40). In agreement with reported IC_50_ values, and in agreement with our hypothesis, T-00127-HEV1 (at 50 μM and 100 μM) attenuates EMCV-mediated MIN6 cell lysis in a concentration-dependent manner (Fig. 3*B*). Much like MIN6 cells, EMCV-mediated lysis of MEF was also attenuated in a similar concentration-dependent manner (Fig. 3*C*). These findings are consistent with a role for PI4KIIIα in EMCV replication and provide a rationale for further investigation.

### siRNA-mediated knockdown of PI4KA, but not PI4KB, attenuates EMCV mRNA accumulation

Previous studies have reported differential utilization of the PI4KIII isoforms for picornavirus replication complex stabilization across different virus types (35, 38, 41). While enteroviruses utilize PI4KIIIβ for host membrane rearrangement and replication complex stabilization (38), it has been suggested that other picornaviruses, such as cardioviruses (EMCV), utilize PI4KIIIα (35). Results from PI4K inhibitor studies (Fig. 3, *B* and *C*), where only high concentrations nearing the IC_50_ for PI4KA decrease EMCV-mediated lysis, support EMCV utilization of PI4KIIIα as the primarily used isoform for replication in β-cells.

**Figure 4:**
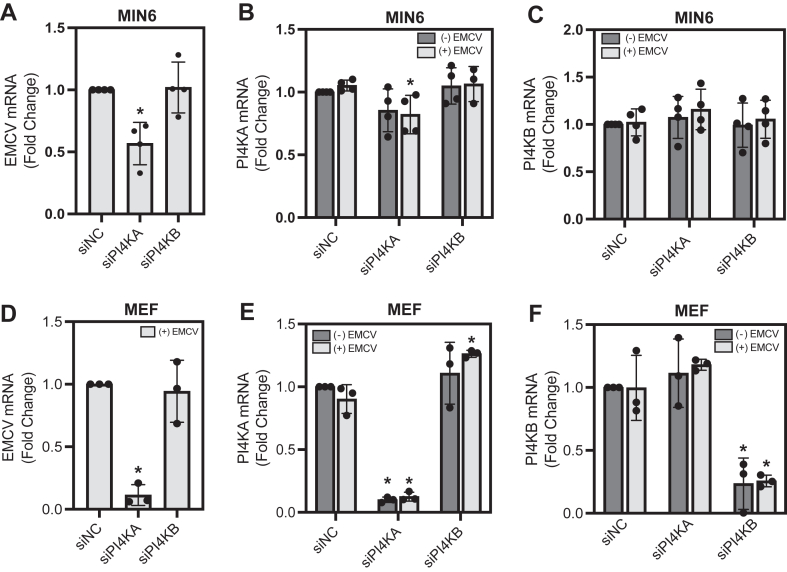
**The effect of PI4K knockdown on EMCV mRNA accumulation.** MIN6 cells (300,000 cells/400 μl, *A*–*C*) or MEF cells (40,000 cells/400 μl, *D*–*F*) were reverse transfected with nontargeting siRNA (NC [negative control]) or siRNA targeting PI4KA or PI4KB, then infected with EMCV, as indicated. EMCV (*A* and *D*), PI4KA (*B* and *E*), and PI4KB (*C* and *F*) mRNA accumulation was measured *via* RT–qPCR 12 h postinfection. Results are the average ± SD of four independent experiments (*A*–*C*) or the average ± SD of three independent experiments (*D*–*F*), with statistically significant differences indicated (∗*p* < 0.05). EMCV, encephalomyocarditis virus; MEF, mouse embryonic fibroblast.

To further examine whether PI4KIIIα is the isoform used during EMCV infection in β-cells, and to explore if compensation from other isoforms occurs, PI4KA and PI4KB were targeted for siRNA-mediated knockdown. In general, MIN6 cells do not transfect well; however, a modest ∼20% knockdown of PI4KA mRNA results in an ∼45% reduction in EMCV mRNA accumulation in infected MIN6 cells (Fig. 4, *A* and *B*). Appreciable knockdown of PI4KB was not achieved in MIN6 cells (Fig. 4*C*). In MEF, an ∼90% knockdown of PI4KA and PI4KB was achieved (Fig. 4, *E* and *F*, respectively), and in agreement with inhibitor data, knockdown of PI4KA, but not PI4KB, resulted in a significant reduction of EMCV mRNA accumulation (Fig. 4*C*). These data support PI4KIIIα as the isoform utilized for efficient viral replication during EMCV infection. In addition, these results support exclusive utilization of PI4KIIIα for EMCV replication.

### NO attenuates PI4KIIIα-mediated PI4P accumulation selectively in MIN6 cells

**Figure 5:**
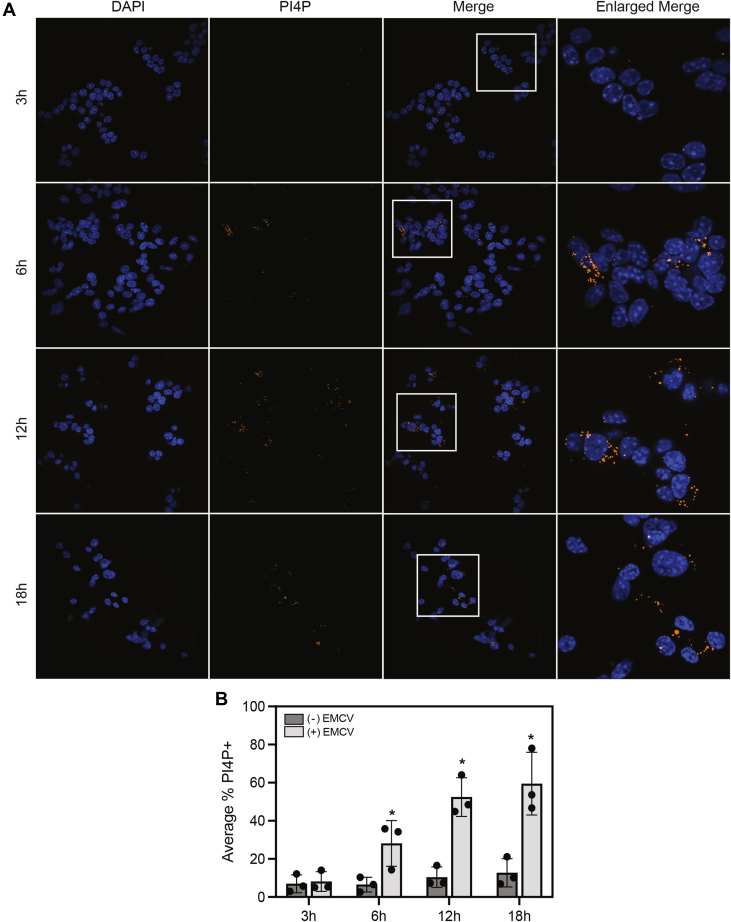
**Time-dependent accumulation of PI4P in EMCV-infected MIN6 cells.** MIN6 cells (600,000 cells/800 μl) were infected with 5 MOI EMCV for 3 to 18 h (as indicated), then PI4P accumulation was identified by immunofluorescence staining (*orange*). Nuclei were identified using DAPI fluorescence (*blue*). Cells were visualized at 40× magnification (*A*). Cells with accumulation of PI4P puncta were manually quantified using the cell counter feature from Fiji ImageJ2 (*B*). Results are representative (*A*) of three independent experiments and the average ± SD of three independent experiments (*B*). Statistically significant differences are indicated (∗*p* < 0.05). DAPI, 4′,6-diamidino-2-phenylindole; EMCV, encephalomyocarditis virus; MOI, multiplicity of infection; PI4P, phosphatidylinositol-4-phosphate.

The product of PI4KIIIα, PI4P, is one of the most highly abundant phosphoinositide phosphates found in cells (Fig. S1) (31, 32). Following EMCV infection, PI4P accumulates into highly concentrated puncta (Fig. 5*A*). These findings are consistent with the notion that PI4KIIIα function is hijacked during EMCV infection, and PI4P production is directed predominantly to the ER membrane (34, 35). It is important to note that because of the cellular abundance of this phospholipid, the display range, which is the minimum and maximum intensity value of a fluorescent signal, was uniformly adjusted to correct for basal PI4P fluorescence. This approach allows for full visualization of PI4P accumulates directly associated with EMCV replication (Fig. S1). The accumulation of PI4P puncta (*orange*) was observed as early as 6 h post-EMCV infection and persists for up to 18 h (Fig. 5, *A* and *B*). Because maximal PI4P accumulation was observed 12 h post-EMCV infection in MIN6 cells, this time point was used for subsequent studies.

**Figure 6:**
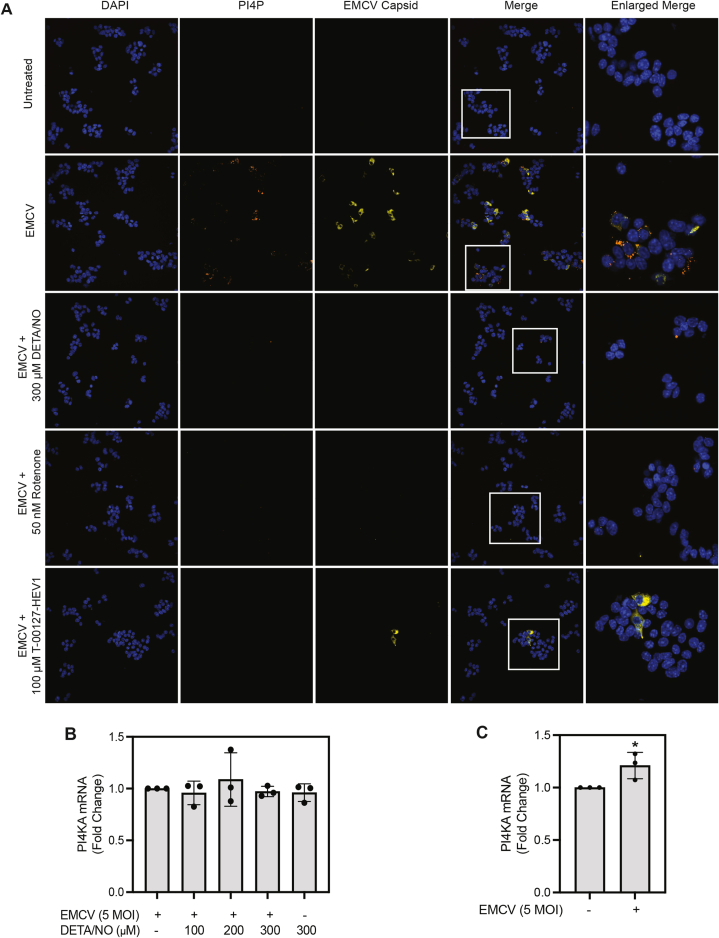
**The effect of nitric oxide on PI4P lipid accumulation in EMCV-infected MIN6 cells.** MIN6 cells (600,000 cells/800 μl) were infected with 5 MOI EMCV with or without 300 μM DETA/NO, 50 nM rotenone, or 100 μM T-00127-HEV1 for 12 h (*A*). Nuclei were identified using DAPI fluorescence (*blue*), and cells containing EMCV (*yellow*) and PI4P (*orange*) were identified by immunofluorescence microscopy at 40× magnification. The effects of nitric oxide (*B*) and EMCV infection (*C*) on PI4KA expression were measured by RT–qPCR 12 h postinfection and normalized to EMCV-treated samples (*B*) or the untreated control (*C*). Results are representative of (*A*) or the average ± SD (*B* and *C*) of three independent experiments. Statistically significant differences are indicated (∗*p* < 0.05). DAPI, 4′,6-diamidino-2-phenylindole; DETA/NO, (Z)-1-(*N*-(2-aminoethyl)-*N*-(2-ammonioethyl)amino)diazen-1-ium-1,2-diolate; EMCV, encephalomyocarditis virus; MOI, multiplicity of infection; PI4P, phosphatidylinositol-4-phosphate.

Knowing that NO inhibits PI3K activity when present at iNOS-derived, micromolar concentrations (Fig. 2), the effects of DETA/NO on PI4P accumulation in EMCV-infected MIN6 cells were examined. As shown in Figure 6*A*, DETA/NO attenuates the accumulation of PI4P puncta (*orange*) and EMCV capsid protein (*yellow*) following a 12-h EMCV infection (Fig. 6*A*). These data are consistent with the effects of the PI4K inhibitor, T-00127-HEV1, and support a role for PI4KIIIα as a potential target for the inhibitory actions of NO on EMCV replication (Fig. 6*A*). We also show that neither DETA/NO nor EMCV infection substantially modifies PI4KA mRNA expression in MIN6 cells, suggesting that PI4KIIIα activity is likely being affected by the actions of NO (Fig. 6, *B* and *C*, respectively).

In previous studies, we have shown that the inhibitory actions of NO on EMCV replication are β-cell-selective and associated with an inhibition of oxidative mitochondrial metabolism (23, 29). Exposure to iNOS-derived levels of NO decreases cellular ATP levels in islets, fluorescence-activated cell sorting–purified primary β-cells, and mouse insulinoma cell lines by ∼8- to 10-fold (19, 20, 42). In agreement with previous studies, inhibition of complex I of the electron transport chain, using rotenone, attenuates PI4P accumulation and EMCV capsid protein accumulation to levels similar to the actions of DETA/NO and T-00127-HEV1 in MIN6 cells (Fig. 6*A*).

### The effects of NO on the early stages of the EMCV life cycle

**Figure 7:**
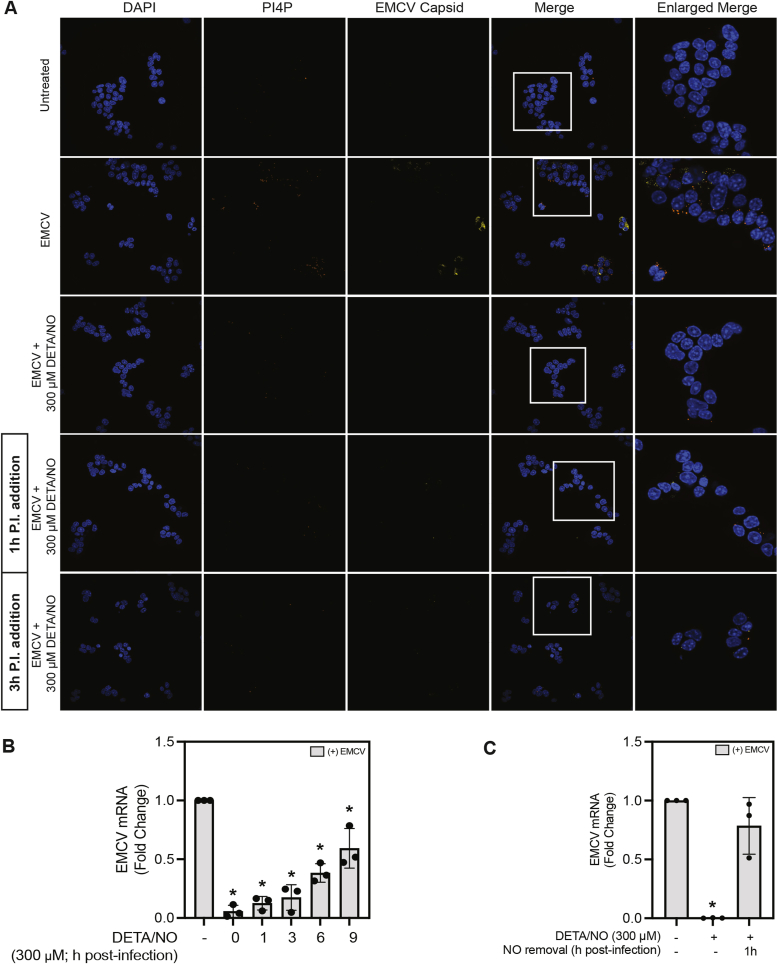
**Stage in the EMCV life cycle that is sensitive to nitric oxide.** MIN6 cells (600,000 cells/800 μl) were infected with 5 MOI EMCV for 12 h with or without 300 μM DETA/NO added at the time of infection, 1 h postinfection, or 3 h postinfection. EMCV capsid protein (*yellow*) and PI4P (*orange*) were identified by immunostaining for EMCV capsid protein and PI4P, and visualized using immunofluorescence microscopy. Nuclei were identified using DAPI fluorescence (*blue*), and cells were visualized at 60× (oil) magnification (*A*). MIN6 cells (300,000 cells/400 μl) were infected with 5 MOI EMCV with or without 300 μM DETA/NO added at the time of infection, or at indicated times postinfection, and EMCV (viral protein-1 [VP1]) mRNA accumulation was measured by RT–qPCR 12 h postinfection (*B*). MIN6 cells (300,000 cells/400 μl) were infected with 5 MOI EMCV with or without 300 μM DETA/NO for 12 h. One hour following infection, media were replaced, and DETA/NO was either added back to culture media or removed, as indicated. EMCV VP1 mRNA accumulation was measured by RT–qPCR (*C*). Results are representative of three independent experiments (*A*) or the average ± SD of three independent experiments (*B* and *C*). Statistically significant differences are indicated (∗*p* < 0.05). DAPI, 4′,6-diamidino-2-phenylindole; DETA/NO, (Z)-1-(*N*-(2-aminoethyl)-*N*-(2-ammonioethyl)amino)diazen-1-ium-1,2-diolate; EMCV, encephalomyocarditis virus; PI4P, phosphatidylinositol-4-phosphate.

To determine if early stages in EMCV infection (viral entry, uncoating, initial translation, or polyprotein cleavage) are sensitive to the actions of NO, MIN6 cells were infected with EMCV, and DETA/NO was added at the time of infection, or various times postinfection. EMCV infection resulted in a robust accumulation of PI4P (*orange*) and EMCV capsid protein (*yellow*, Fig. 7*A*). When MIN6 cells were infected with EMCV and treated with DETA/NO at the time of infection, PI4P and EMCV capsid protein accumulation were prevented (Fig. 7*A*). These findings are consistent with the results presented in Figure 6*A*. In EMCV-infected MIN6 cells treated with DETA/NO administered 1 h and 3 h postinfection, PI4P and EMCV capsid protein accumulation are attenuated to levels comparable to those observed when DETA/NO is added at the time of infection (Fig. 7*A*). Since NO attenuates virus replication whether added at the time of infection, or up to 3 h postinfection, these data suggest that early events in the EMCV life cycle are not modified by the actions of NO.

In agreement with our imaging studies, when DETA/NO is added 1 or 3 h postinfection, EMCV mRNA accumulation was nearly completely attenuated (Fig. 7*B*), similar to samples treated with DETA/NO at the time of infection. When DETA/NO was added 6 or 9 h postinfection, a ∼50% decrease in EMCV mRNA accumulation was observed (Fig. 7*B*). Attenuation observed as late as 6 and 9 h postinfection precludes early events in the EMCV life cycle from being possible targets of the actions of NO. In further support for this conclusion, we show that NO does not modify the viral capsid or prevent viral entry, as inhibition of EMCV mRNA accumulation (12 h postinfection) is not observed when DETA/NO is added during infection and then removed by washing 1 h postinfection (Fig. 7*C*). These findings show that early events in the viral life cycle (virus entry/internalization, uncoating, initial translation, or polyprotein cleavage), or events that occur prior to PI4P accumulation, are not modified by NO-mediated mitochondrial inhibition.

### NO inhibits PI4P accumulation and EMCV replication in mouse islet cells

**Figure 8:**
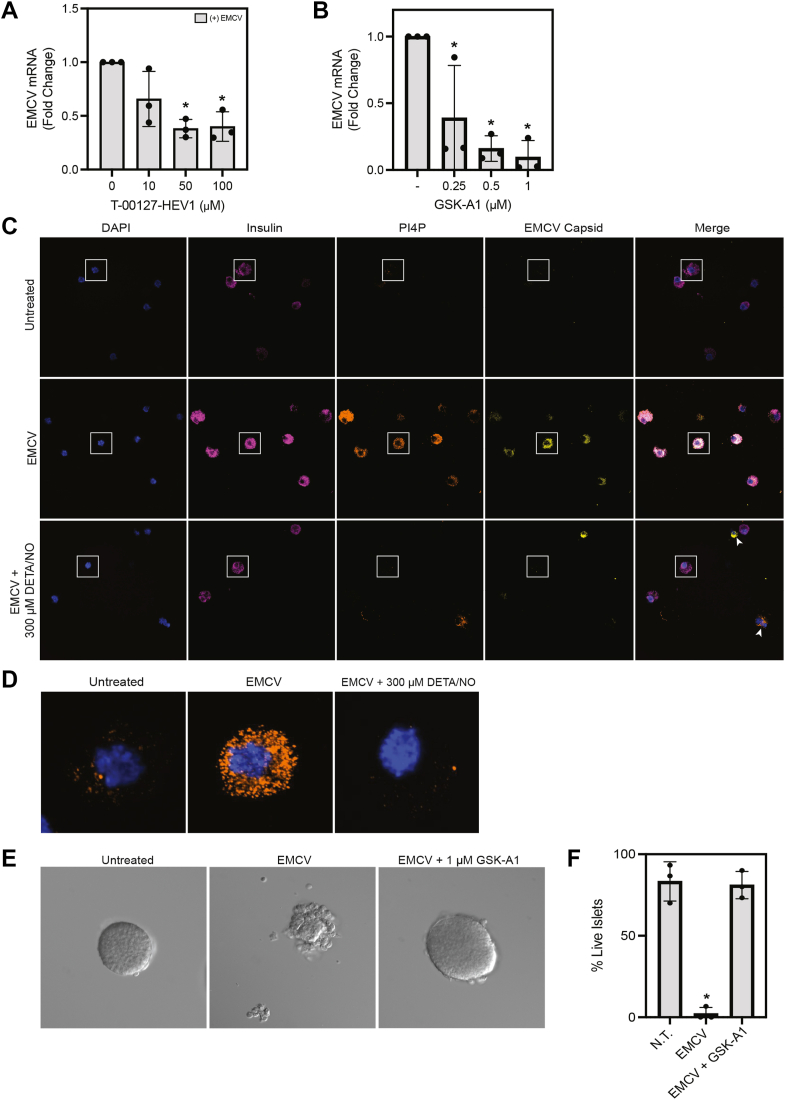
**DETA/NO attenuates EMCV replication by limiting PI4P accumulation in primary mouse β-cells.** Mouse islets (85 islets/condition) were plated and infected with 5 MOI EMCV with or without indicated concentrations of T-00127-HEV1 (*A*) or GSK-A1 (*B*). EMCV (viral protein-1 [VP1]) mRNA was quantified by RT–qPCR 12 h postinfection (*A* and *B*). Mouse islet cells (85,000–100,000 cells/condition) were dispersed, plated, then infected with 5 MOI EMCV with or without 300 μM DETA/NO. Nuclei (DAPI fluorescence, *blue*), β-cells (insulin, *magenta*), EMCV capsid protein (*yellow*), and PI4P (*orange*) were visualized by immunofluorescence microscopy at 60× (oil objective) magnification (*C*). Images identified by *boxes* outlined in *white* were enlarged to allow for enhanced visualization of PI4P accumulation (*D*). Thirty islets/condition were counted into a 12-well optical plate. Islets were infected with 5 MOI EMCV, and treated with or without GSK-A1. DIC images were taken prior to and every 24 h during infection to observe islet structural integrity. Following the termination of the infection (72 h), islet viability was evaluated by manual counting in a blinded manner (*E* and *F*). Results are an average ± SD of three independent experiments (*A*, *B*, and *F*) or representative of three independent experiments (*C*–*E*). Statistically significant differences are indicated (∗*p* < 0.05). DAPI, 4′,6-diamidino-2-phenylindole; DETA/NO, (Z)-1-(*N*-(2-aminoethyl)-*N*-(2-ammonioethyl)amino)diazen-1-ium-1,2-diolate; DIC, differential interference contrast; EMCV, encephalomyocarditis virus; PI4P, phosphatidylinositol-4-phosphate.

The pancreatic islet represents a heterogeneous population of endocrine and nonendocrine cells, of which β-cells comprise ∼70% of the endocrine islet. To determine whether NO attenuates PI4P accumulation in primary mouse β-cells, islets isolated from C57BL/6 mice were infected with EMCV and treated with or without increasing concentrations of the PI4K inhibitor, T-00127-HEV1. In agreement with insulinoma cell line data, T-00127-HEV1, at concentrations that attenuate EMCV mRNA accumulation in MIN6 cells, also attenuates EMCV mRNA accumulation in primary islet cells (Fig. 8*A*). Upon identification of a commercially available PI4KIIIα-selective inhibitor, GSK-A1, these studies were extended to confirm our findings. Treatment of mouse islets with increasing concentrations of GSK-A1 resulted in attenuation of EMCV mRNA accumulation (Fig. 8*B*). Taken together, these findings confirm a role for PI4KIIIα, but not PI4KIIIβ, in EMCV replication in mouse β-cells.

To evaluate the actions of NO on PI4P accumulation, islets isolated from C57BL/6 mice were dispersed into single cells and then infected with EMCV in the presence or absence of DETA/NO. Much like the response observed in MIN6 cells, PI4P (*orange*) accumulated in both insulin-containing β-cells (identified by insulin-staining, *magenta*) and non–insulin-containing cells 12 h post-EMCV infection (Fig. 8, *C* and *D*). PI4P accumulation was observed in cells also expressing the EMCV capsid protein (*yellow*) (Fig. 8*C*). In EMCV-infected islet cells treated with DETA/NO, PI4P lipid and EMCV capsid protein accumulation were attenuated in insulin-containing cells (Fig. 8*C*), whereas DETA/NO did not modify the accumulation of PI4P or EMCV capsid protein in non–insulin-containing cells (Fig. 8*C*, *arrows*). These findings indicated that, like insulinoma cells, PI4P accumulation is attenuated by NO in primary mouse β-cells. Further, these data support selectivity for β-cells since PI4P and EMCV capsid accumulation persists in non–insulin-containing cells in the presence of NO.

To further support these findings, we sought to determine if the inhibition of PI4KIIIα not only inhibits PI4P accumulation and EMCV replication, but also that islet viability is preserved following EMCV infection. Islet viability was monitored over a 48 to 72 h period of EMCV infection by differential interference contrast (DIC) microscopy. Mouse islets were treated with or without GSK-A1 during infection, and viability was determined by trypan blue staining at the termination of the infection (43, 44, 45). Following EMCV infection, islets were mostly disintegrated, and very few of the remaining intact islets were viable (Fig. 8, *E* and *F*). Islets treated with GSK-A1 during EMCV infection remained nearly fully intact and viable (Fig. 8, *E* and *F*), further confirming a role for PI4KIIIα in EMCV infection.

## Discussion

In response to a viral infection, circulating immune cells release inflammatory cytokines into the bloodstream, and islet-resident macrophages can release cytokines in the islet microenvironment (18, 46). It is well known that islet exposure to cytokines results in an inhibition of insulin secretion by β-cells (18, 47). The inhibitory actions of cytokines on insulin secretion are mediated by the expression of iNOS, and production of low micromolar levels of NO (18, 19, 20, 21). NO is a potent mitochondrial inhibitor that decreases the activity of aconitase (TCA cycle) and complex IV of the electron transport chain, inhibiting mitochondrial oxidative metabolism (19, 24, 25). While most cell types can utilize anaerobic glycolysis to maintain ATP levels, due, in part, to low-level expression of lactate dehydrogenase, β-cells are not capable of replenishing NAD^+^ to levels necessary for sustained glycolysis (GAPDH requires NAD^+^) (26, 27). As a result, cellular ATP levels are depleted in β-cells (19, 20). Increased cellular ATP (ATP/ADP ratio) is required for K^+^_ATP_ channel closure, β-cell depolarization, Ca^2+^ entry, and Ca^2+^-dependent insulin secretion, meaning that the inhibition of oxidative metabolism by NO effectively shuts down insulin secretion (19, 20, 48). Since NO inhibits the primary function of β-cells, the actions of cytokines have historically been viewed as damaging.

Recently, we have identified physiological benefits for the endogenous production of NO by β-cells, particularly during a viral infection (23, 29). We have shown that exogenous addition of NO, using chemical donors at physiological concentrations, results in the inhibition of EMCV mRNA accumulation and EMCV-mediated β-cell lysis (23). Importantly, the actions of NO are selective for β-cells and are associated with a depletion in cellular ATP levels (29). Knowing that NO inhibits aconitase (TCA cycle) and complex IV of the electron transport chain, we have shown that other inhibitors of mitochondrial oxidative metabolism (rotenone, FCCP, and antimycin A) also attenuate EMCV mRNA accumulation and EMCV-mediated insulinoma cell lysis (29). Much like NO, mitochondrial inhibitors do not attenuate EMCV mRNA accumulation or lysis of non–β-cells, as these cells have the capacity to maintain cellular ATP levels in response to mitochondrial inhibition *via* glycolytic metabolism (27, 29). These exciting findings show that the mechanism responsible for the inhibition of insulin secretion by NO also functions to protect β-cells during a viral infection. The goal of the current study was to define the mechanism(s) by which NO-mediated ATP depletion attenuates EMCV-mediated lysis selectively in β-cells.

In studying the concentration-dependent actions of DPTA/NO on PI3K signaling, we made the surprising observation that low concentrations (<200 μM) of DPTA/NO stimulate, whereas high concentrations (>200 μM) of DPTA/NO attenuate phosphorylation of the PI3K substrate, Akt (Fig. 2, *A* and *B*). These observations were not observed in non–β-cells (Fig. 2, *C* and *D*). The concentration-dependent actions of NO on PI3K signaling agree with our recent studies showing that there is a narrow concentration range of NO that controls gene expression in β-cells (49). At concentrations that do not inhibit mitochondrial oxidation (∼0.5–1 μM), NO stimulates the expression of genes that have been shown to protect β-cells from oxidative damage. However, at concentrations above ∼1 μM, NO fails to stimulate expression of these genes. These responses are observed under a narrow concentration range of 100 μM and 200 μM DPTA/NO (∼0.5–1 μM NO), with the loss of gene expression associated with an inhibition of mitochondrial oxidative metabolism (49).

Since inhibition of PI3K did not modify EMCV replication in β-cells, we explored whether other members of the PI3/4K family may be inhibited by NO at concentrations that limit EMCV replication. PI4KIIIα is a host factor that participates in the replication of several picornaviruses (including EMCV and other cardioviruses). During infection, PI4P production is directed to the ER membrane, where it is used as an anchor to stabilize viral replication complexes. This is an essential step for efficient viral replication initiation (Fig. 3*A*). Using PI4K inhibitors, T-00127-HEV1 and GSK-A1, as well as siRNA-mediated depletion approaches, we provide evidence that the depletion and loss of activity of PI4KIIIα, but not PI4KIIIβ, attenuates EMCV replication (Figs. 3, 4 and 8). Additionally, we show that the selective inhibition of PI4KIIIα activity by GSK-A1 largely prevents EMCV-mediated islet degeneration and cell death (Fig. 8, *E* and *F*). Much like the PI3/4K family member, PI3K, concentrations of NO that inhibit mitochondrial oxidative metabolism (Fig. 2), attenuate PI4KIIIα activity. More specifically, NO, when provided at iNOS-derived levels, attenuates PI4P and EMCV capsid protein accumulation in β-cells (Fig. 6*A*). Consistent with our previous studies (23), inhibitors of mitochondrial oxidative metabolism, such as rotenone (inhibitor of complex I of the electron transport chain), also attenuate PI4P and EMCV capsid accumulation (Fig. 6*A*).

The EMCV life cycle includes several steps that take place over a 16- to 25-h period (14). Early events in the viral life cycle include viral entry/internalization, initial translation, polyprotein cleavage, and replication (14). To evaluate early stages in the EMCV life cycle as potential targets for the actions of NO, MIN6 cells were treated with DETA/NO 1 and 3 h post-EMCV infection (time points that are aligned with early events in the EMCV life cycle). Importantly, NO was equally effective at inhibiting PI4P and EMCV capsid accumulation when added to insulinoma cells 1 and 3 h postinfection, suggesting that NO does not modify viral entry/internalization or initial translation (Fig. 7, *A* and *B*).

While NO has been shown to inhibit viral protease activity, *via* S-nitrosation of an active site cysteine for some members of the picornavirus family (50, 51, 52), we do not believe that inhibition of polyprotein cleavage is responsible for the observations made in these studies. In support of this conclusion, mitochondrial toxins, such as rotenone, attenuate PI4P accumulation and EMCV replication (Fig. 6*A*). In addition, the inhibitory actions of NO and mitochondrial toxins on EMCV replication are selective for β-cells (23, 29). If the observed attenuation of PI4P accumulation was due to S-nitrosation, it would be expected to occur in all cell types (Fig. 8*B*), and other inhibitors of mitochondrial oxidative metabolism would not modify EMCV replication (Fig. 6*A*).

While we have identified the loss of PI4P accumulation as a target for the actions of NO, it is not clear if NO, or the resulting loss of cellular ATP, acts directly on PI4K activity. The *K*_*m*_ for ATP of PI4KA is 209 μM (53), and while inhibition of mitochondrial metabolism by NO, or other mitochondrial inhibitors, decreases β-cell ATP levels by ∼8- to 10-fold in INS832/13 cells (rat insulinoma cell line) and rat islets, mitochondrial inhibition depletes ATP levels by ∼50% in MIN6 cells (29, 54, 55). Since basal levels of ATP are 1 to 2 mM, we do not expect cellular ATP to be depleted to levels that modify PI4KIIIα activity in MIN6 cells. It is possible that the inhibitory actions of NO on PI4P accumulation (*via* inhibition of PI4KIIIα) are indirect and may reflect changes (accumulation or loss) in levels of metabolites that limit PI4KIIIα activity during EMCV infection.

The β-cell response to endogenously produced NO (following cytokine exposure) has historically been thought to be detrimental to β-cell health, as it mediates the inhibitory actions of cytokines on mitochondrial oxidative metabolism and insulin secretion (18, 19, 20, 21, 22, 47). In recent studies, we have shown that NO places β-cells in a state of “metabolic suspended animation,” where oxidative metabolism is inhibited in a reversible manner (24, 28, 55, 56). When in this state, β-cells are resistant to cell death, as caspase activation and virus replication are inhibited (23, 29). We now show that the mechanism by which NO, and mitochondrial toxins, attenuates EMCV replication selectively in β-cells is through the inhibition of PI4KIIIα activity. This inhibition limits PI4P accumulation at the ER membrane, a required event for efficient EMCV replication. Together, these studies provide additional evidence in support of our hypothesis that NO and cytokine signaling in the endocrine islet is physiological, and functions to protect β-cells from pathogens, such as viruses.

## Experimental procedures

### Materials and animals

Male C57BL/6 mice were purchased from The Jackson Laboratory. Mice were housed in the MCW Biomedical Resource Center. The Institutional Animal Care and Use Committees at the Medical College of Wisconsin approved all animal use and experimental procedures used in these studies. MIN6 cells were obtained from AddexBio. MEF and Eagle’s minimum essential medium were obtained from the American Type Culture Collection (ATCC). Dulbecco’s modified Eagle’s medium (DMEM), Connaught Medical Research Laboratories 1066 medium, and sodium pyruvate were purchased from ThermoFisher. NO donors DETA/NO and DPTA/NO were purchased from Cayman Chemical. NO donors were dissolved in 10 mM NaOH. Rotenone was purchased from MilliporeSigma. T-00127-HEV1 (PI4KIIIβ-selective inhibitor) and GSK-A1 (PI4KIIIα-selective inhibitor) were purchased from MedChemExpress and were dissolved in dimethyl sulfoxide. Primary antibodies and their respective sources are as follows: mouse anti-GAPDH (AM4300) was purchased from ThermoFisher, rabbit-anti-Mengo (EMCV capsid) (a generous gift from Dr Ann Palmenberg, University of Wisconsin-Madison, Madison, WI), rabbit-anti-phospho-Akt (S473) (Cell Signaling Technology), rabbit-anti-(pan) Akt (Cell Signaling Technology), mouse-anti-PI4P (Echelon Biosciences), and guinea pig–anti-swine insulin (DAKO). Secondary antibodies and their respective sources are as follows: horseradish peroxidase (HRP)–conjugated donkey–anti-mouse (Jackson ImmunoResearch Laboratories), HRP-conjugated donkey–anti-rabbit (Jackson ImmunoResearch Laboratories), CY3-conjugated AffiniPure donkey–anti-guinea pig IgG (Jackson ImmunoResearch Laboratories), CY3-conjugated AffiniPure donkey–anti-mouse IgG (Jackson ImmunoResearch Laboratories), AlexaFlour488-conjugated donkey–anti-rabbit IgG (Invitrogen), AlexaFlour488-conjugated donkey–anti-mouse IgG (Fisher Scientific), and AlexaFlour647 donkey–anti-rabbit IgG (Invitrogen).

### Cell culture and primary islet isolation

MIN6 cells (AddexBio) were maintained in DMEM containing 15% heat-inactivated fetal bovine serum, l-glutamine, sodium pyruvate, and β-mercaptoethanol. MEFs (ATCC) were maintained in DMEM containing 10% heat-inactivated fetal bovine serum, l-glutamine, sodium pyruvate, and Hepes. Cells were removed from growth flasks by incubating in 0.05% trypsin in 0.53 mM EDTA at 37 °C for 2 to 5 min. Cells were grown at 37 °C and 5% CO_2_ for at least 10 h (MIN6) or 5 h (MEF) prior to the initiation of an experiment. Islets from adult male C57BL/6 mice were isolated and cultured according to a previously described protocol (57, 58, 59). Islets were either infected intact or dispersed into single cells prior to infection. Islet dispersion was performed as previously described (56, 57). Dispersed islet cells were incubated at 37 °C and 5% CO_2_ for at least 6 h prior to infection.

### EMCV propagation and infection

EMCV stocks were a generous gift from Dr Ji-Won Yoon (University of Calgary, Calgary, Alberta, Canada). EMCV variants have been previously described (60). EMCV stocks were propagated, and plaque assays were performed as previously described (29). MIN6 and MEF cells were grown in monolayers and infected at a multiplicity of infection (MOI) of 5 and 0.1, respectively, by direct addition of EMCV to the culture medium. Mouse islets were infected with 5 MOI of EMCV *via* direct addition to culture medium. Cells were incubated at 37 °C for 1 h, then media were replaced, and the culture was continued for the indicated times.

### Real-time PCR

The RNeasy Mini Kit (Qiagen) was used according to the manufacturer’s instructions to isolate total RNA from cell lysates. DNase digestion was completed using the Turbo DNA-free procedure (Applied Biosystems). First-strand complementary DNA synthesis was subsequently performed using the oligo(dT) (Integrated DNA Technologies [IDT]) and Maxima H-minus reverse transcriptase (ThermoFisher) according to the manufacturer’s instructions. SsoFast Evagreen Supermix (Bio-Rad) was used to perform quantitative real-time PCR (RT-qPCR). Samples were analyzed, and results were obtained from the Bio-Rad CFX96 Real-Time detection system or the Bio-Rad CFX Duet Real-Time detection system, according to the manufacturer’s instructions. Samples were normalized to GAPDH (ΔC_t_) and expressed as fold change relative to control samples (2^-ΔΔCt^). Primers were purchased from IDT, and the sequences are listed in Table S1.

### siRNA-mediated knockdown

A negative control siRNA and siRNAs against either mouse PI4KA or mouse PI4KB were purchased from IDT. Each siRNA was reverse transfected into MIN6 or MEF cells at a final concentration of 100 nM using Opti-MEM reduced serum medium (ThermoFisher) and Lipofectamine 2000 (ThermoFisher). MEFs were transfected for 18 h, immediately followed by EMCV infection/treatment. MIN6 cells were transfected for 18 h, after which the media were replaced, and the cells were cultured for an additional 24 h prior to infection/treatment. The efficiency of PI4KA and PI4KB knockdown was measured by RT–qPCR. siRNA sequences are listed in Table S2.

### Western blot analysis

Cells were washed once with PBS and lysed with Laemmli sample buffer. Proteins were separated by SDS-PAGE, transferred to a nitrocellulose, and membranes were blocked in 3% bovine serum albumin (BSA) in Tris-buffered saline with Tween-20 (TBST) for 1 h, followed by overnight incubation with primary antibody at 4 °C according to the following antibody solutions: rabbit-anti-p-Akt (S473) (1:1000 dilution), rabbit-anti-Akt (1:1000 dilution), EMCV capsid (1:1000 dilution), and GAPDH (1:10,000 dilution). Membranes were washed three times with TBST for 5 min. Membranes were subsequently incubated in either donkey–anti-mouse (HRP conjugated) or donkey–anti-rabbit (HRP conjugated) secondary antibody solutions diluted at 1:20,000 in 1% BSA in TBST at room temperature for 1 h. Immunoreactivity was detected using chemiluminescence (61). Total Akt and GAPDH were used as loading controls.

### Cell viability assay

SYTOX Green nucleic acid staining (Invitrogen) was used to determine cell death. Following infection/treatment, SYTOX Green was added to each well at a final concentration of 5 μM. Digitonin (120 μM) was added to duplicate wells to determine complete cell lysis (28). Cells were incubated for 10 min at 37 °C, followed by fluorescence reading at 504 nm/523 nm excitation/emission. Percent cell death was calculated by normalization of sample fluorescence to the fluorescence of each respective SYTOX Green + digitonin duplicate wells (set as 100% cell death).

### Microscopy studies

#### Immunofluorescence

CellVis 12-well Optical Plates (22.0 mm well size, #1.5P cover glass) were coated with extracellular matrix coating media (DMEM, 2 μg/ml fibronectin, with 1% extracellular matrix gel added immediately prior to plating) and allowed to incubate for 2 to 24 h prior to plating MIN6 cells. Dispersed mouse islet cells were washed twice with PBS, collected by centrifugation and resuspended in 1% BSA in PBS. Islet cells were then centrifuged onto SuperFrost Plus glass microscope slides (Electron Microscopy Sciences) using the Shandon Cytospin II (ThermoFisher Scientific). Samples were fixed in 4% paraformaldehyde for 15 min, permeabilized in 0.2% Triton X-100 in PBS for 20 min, then blocked in 1% BSA in PBS with 0.1% Triton X-100 and 0.25 M glycine for 1 h. Samples were then incubated with EMCV capsid and PI4P primary antibodies (1:1000 dilution in 1% BSA in PBS with 0.05% Triton X-100) at 4° with gentle agitation overnight (12–18 h). Primary mouse islet cell samples included an insulin primary antibody (in addition to EMCV and PI4P, listed previously), used at 1:1000 overnight (without gentle agitation). Following primary antibody incubation, samples were washed three times with PBS, then incubated with CY3-conjugated AffiniPure donkey anti–mouse IgG, CY3-conjugated AffiniPure donkey–anti-guinea pig IgG, AlexaFlour488-conjugated donkey–anti-mouse IgG, AlexaFlour488-conjugated donkey–anti-rabbit IgG secondary antibody, and/or AlexaFlour647-conjugated donkey–anti-rabbit IgG at a dilution of 1:1000 in 1% BSA in PBS for 1 h at room temperature (shielded from light). 4′,6-Diamidino-2-phenylindole staining (5 min at room temperature, shielded from light) was used for nuclear identification. ProLong Gold AntiFade Mounting Reagent (Invitrogen) was used for coverslip mounting. Images were captured using a spinning disc confocal microscope (Nikon Eclipse Ti2-E microscope) equipped with a Yokogawa confocal scanner unit (CSU-W1) at 40× and 60× (oil) magnification. Images were processed using Fiji ImageJ, version 2.1.0. PI4P quantification was conducted using the Cell Counter feature in Fiji ImageJ and manual counting.

#### DIC microscopy

Thirty islets were added per well to a CellVis 12-well Optical Plate. DIC images were captured on the Nikon Eclipse Ti2-E microscope at 20× magnification prior to infection and every 24 h during infection. Following the conclusion of the infection, trypan blue was added to each well to allow identification of dead cells. A lab member, blinded from treatment groups, performed manual counting using a brightfield microscope at 10× magnification, as previously described (43, 44, 45).

### Statistics

Statistical analysis was completed using either a one-way or two-way ANOVA. Significant difference between groups was determined using the Tukey *post hoc* test. Statistical significance (*p* < 0.05) is indicated by an asterisk (∗).

## Data availability

All data not contained in the article will be shared upon request to John A. Corbett, Medical College of Wisconsin, jcorbett@mcw.edu.

## Supporting information

This article contains supporting information.

## Conflict of interest

The authors declare that they have no conflicts of interest with the contents of this article.

## Supporting information

Sup fig leg

## References

[1] Gepts W. (1965). Pathologic anatomy of the pancreas in juvenile diabetes mellitus. Diabetes.

[2] Atkinson M.A., Mirmira R.G. (2023). The pathogenic "symphony" in type 1 diabetes: a disorder of the immune system, β cells, and exocrine pancreas. Cell Metab..

[3] Hyttinen V., Kaprio J., Kinnunen L., Koskenvuo M., Tuomilehto J. (2003). Genetic liability of type 1 diabetes and the onset age among 22,650 young Finnish twin pairs: a nationwide follow-up study. Diabetes.

[4] Redondo M.J., Rewers M., Yu L., Garg S., Pilcher C.C., Elliott R.B. (1999). Genetic determination of islet cell autoimmunity in monozygotic twin, dizygotic twin, and non-twin siblings of patients with type 1 diabetes: prospective twin study. BMJ.

[5] Yoon J.W. (1990). The role of viruses and environmental factors in the induction of diabetes. Curr. Top Microbiol. Immunol..

[6] Patterson C.C., Gyürüs E., Rosenbauer J., Cinek O., Neu A., Schober E. (2012). Trends in childhood type 1 diabetes incidence in Europe during 1989-2008: evidence of non-uniformity over time in rates of increase. Diabetologia.

[7] Jaeckel E., Manns M., Von Herrath M. (2002). Viruses and diabetes. Ann. N. Y. Acad. Sci..

[8] Richardson S.J., Rodriguez-Calvo T., Laiho J.E., Kaddis J.S., Nyalwidhe J.O., Kusmartseva I. (2025). Joint analysis of the nPOD-Virus Group data: the association of enterovirus with type 1 diabetes is supported by multiple markers of infection in pancreas tissue. Diabetologia.

[9] Rodriguez-Calvo T., Laiho J.E., Oikarinen M., Akhbari P., Flaxman C., Worthington T. (2025). Enterovirus VP1 protein and HLA class I hyperexpression in pancreatic islet cells of organ donors with type 1 diabetes. Diabetologia.

[10] Hober D., Alidjinou E.K. (2013). Enteroviral pathogenesis of type 1 diabetes: queries and answers. Curr. Opin. Infect. Dis..

[11] Richer M.J., Horwitz M.S. (2009). Coxsackievirus infection as an environmental factor in the etiology of type 1 diabetes. Autoimmun. Rev..

[12] Yeung W.C., Rawlinson W.D., Craig M.E. (2011). Enterovirus infection and type 1 diabetes mellitus: systematic review and meta-analysis of observational molecular studies. BMJ.

[13] Stene L.C., Oikarinen S., Hyöty H., Barriga K.J., Norris J.M., Klingensmith G. (2010). Enterovirus infection and progression from islet autoimmunity to type 1 diabetes: the Diabetes and Autoimmunity Study in the Young (DAISY). Diabetes.

[14] Carocci M., Bakkali-Kassimi L. (2012). The encephalomyocarditis virus. Virulence.

[15] Shaheen Z.R., Corbett J.A. (2015). Macrophage expression of inflammatory genes in response to EMCV infection. Biomolecules.

[16] Ito M., Yanagi Y., Ichinohe T. (2012). Encephalomyocarditis virus viroporin 2B activates NLRP3 inflammasome. PLoS Pathog..

[17] Zhang W., Huang Z., Huang M., Zeng J. (2020). Predicting severe enterovirus 71-infected hand, foot, and mouth disease: cytokines and chemokines. Mediators Inflamm..

[18] Broniowska K.A., Oleson B.J., Corbett J.A. (2014). β-Cell responses to nitric oxide. Vitam. Horm..

[19] Corbett J.A., Wang J.L., Sweetland M.A., Lancaster J.R., McDaniel M.L. (1992). Interleukin 1 beta induces the formation of nitric oxide by beta-cells purified from rodent islets of Langerhans. Evidence for the beta-cell as a source and site of action of nitric oxide. J. Clin. Invest..

[20] Corbett J.A., McDaniel M.L. (1995). Intraislet release of interleukin 1 inhibits beta cell function by inducing beta cell expression of inducible nitric oxide synthase. J. Exp. Med..

[21] Corbett J.A., Wang J.L., Hughes J.H., Wolf B.A., Sweetland M.A., Lancaster J.R. (1992). Nitric oxide and cyclic GMP formation induced by interleukin 1 beta in islets of Langerhans. Evidence for an effector role of nitric oxide in islet dysfunction. Biochem. J..

[22] Padgett L.E., Broniowska K.A., Hansen P.A., Corbett J.A., Tse H.M. (2013). The role of reactive oxygen species and proinflammatory cytokines in type 1 diabetes pathogenesis. Ann. N. Y. Acad. Sci..

[23] Stafford J.D., Yeo C.T., Corbett J.A. (2020). Inhibition of oxidative metabolism by nitric oxide restricts EMCV replication selectively in pancreatic beta-cells. J. Biol. Chem..

[24] Cleeter M.W., Cooper J.M., Darley-Usmar V.M., Moncada S., Schapira A.H. (1994). Reversible inhibition of cytochrome c oxidase, the terminal enzyme of the mitochondrial respiratory chain, by nitric oxide. Implications for neurodegenerative diseases. FEBS Lett..

[25] Gardner P.R., Costantino G., Szabó C., Salzman A.L. (1997). Nitric oxide sensitivity of the aconitases. J. Biol. Chem..

[26] Sekine N., Cirulli V., Regazzi R., Brown L.J., Gine E., Tamarit-Rodriguez J. (1994). Low lactate dehydrogenase and high mitochondrial glycerol phosphate dehydrogenase in pancreatic beta-cells. Potential role in nutrient sensing. J. Biol. Chem..

[27] Marroquin L.D., Hynes J., Dykens J.A., Jamieson J.D., Will Y. (2007). Circumventing the Crabtree effect: replacing media glucose with galactose increases susceptibility of HepG2 cells to mitochondrial toxicants. Toxicol. Sci..

[28] Oleson B.J., Broniowska K.A., Yeo C.T., Flancher M., Naatz A., Hogg N. (2019). The role of metabolic flexibility in the regulation of the DNA damage response by nitric oxide. Mol. Cell. Biol..

[29] Stafford J.D., Shaheen Z.R., Yeo C.T., Corbett J.A. (2020). Inhibition of mitochondrial oxidative metabolism attenuates EMCV replication and protects β-cells from virally mediated lysis. J. Biol. Chem..

[30] Boura E., Nencka R. (2015). Phosphatidylinositol 4-kinases: function, structure, and inhibition. Exp. Cell Res..

[31] Bura A., Čabrijan S., Đurić I., Bruketa T., Jurak Begonja A. (2023). A plethora of functions condensed into tiny phospholipids: the story of PI4P and PI(4,5)P. Cells.

[32] De Matteis M.A., Wilson C., D'Angelo G. (2013). Phosphatidylinositol-4-phosphate: the golgi and beyond. Bioessays.

[33] Jackson T., Belsham G.J. (2021). Picornaviruses: a view from 3A. Viruses.

[34] Melia C.E., Peddie C.J., de Jong A.W.M., Snijder E.J., Collinson L.M., Koster A.J. (2019). Origins of enterovirus replication organelles established by whole-cell electron microscopy. mBio.

[35] Dorobantu C.M., Albulescu L., Harak C., Feng Q., van Kampen M., Strating J.R.P.M. (2015). Modulation of the host lipid landscape to promote RNA virus replication: the picornavirus encephalomyocarditis virus converges on the pathway used by hepatitis C virus. PLoS Pathog..

[36] Hofmann S., Krajewski M., Scherer C., Scholz V., Mordhorst V., Truschow P. (2018). Complex lipid metabolic remodeling is required for efficient hepatitis C virus replication. Biochim. Biophys. Acta Mol. Cell Biol. Lipids.

[37] Albulescu L., Bigay J., Biswas B., Weber-Boyvat M., Dorobantu C.M., Delang L. (2017). Uncovering oxysterol-binding protein (OSBP) as a target of the anti-enteroviral compound TTP-8307. Antivir. Res..

[38] Dorobantu C.M., van der Schaar H.M., Ford L.A., Strating J.R.P.M., Ulferts R., Fang Y. (2014). Recruitment of PI4KIIIβ to coxsackievirus B3 replication organelles is independent of ACBD3, GBF1, and Arf1. J. Virol..

[39] Arita M., Kojima H., Nagano T., Okabe T., Wakita T., Shimizu H. (2011). Phosphatidylinositol 4-kinase III beta is a target of enviroxime-like compounds for antipoliovirus activity. J. Virol..

[40] Mejdrová I., Chalupská D., Kögler M., Šála M., Plačková P., Baumlová A. (2015). Highly selective phosphatidylinositol 4-Kinase IIIβ inhibitors and structural insight into their mode of action. J. Med. Chem..

[41] Spickler C., Lippens J., Laberge M.K., Desmeules S., Bellavance É., Garneau M. (2013). Phosphatidylinositol 4-kinase III beta is essential for replication of human rhinovirus and its inhibition causes a lethal phenotype in vivo. Antimicrob. Agents Chemother..

[42] Hughes K.J., Meares G.P., Hansen P.A., Corbett J.A. (2011). FoxO1 and SIRT1 regulate beta-cell responses to nitric oxide. J. Biol. Chem..

[43] Hughes K.J., Chambers K.T., Meares G.P., Corbett J.A. (2009). Nitric oxides mediates a shift from early necrosis to late apoptosis in cytokine-treated β-cells that is associated with irreversible DNA damage. Am. J. Physiol. Endocrinol. Metab..

[44] Corbett J.A., McDaniel M.L. (1994). Reversibility of interleukin-1 beta-induced islet destruction and dysfunction by the inhibition of nitric oxide synthase. Biochem. J..

[45] Lacy P.E., Young D.A., Fink C.J. (1968). Studies on insulin secretion in vitro from isolated islets of the rat pancreas. Endocrinology.

[46] Lacy P.E. (1994). The intraislet macrophage and type I diabetes. Mt Sinai J. Med..

[47] Mandrup-Poulsen T., Bendtzen K., Nielsen J.H., Bendixen G., Nerup J. (1985). Cytokines cause functional and structural damage to isolated islets of Langerhans. Allergy.

[48] Rutter G.A., Pullen T.J., Hodson D.J., Martinez-Sanchez A. (2015). Pancreatic β-cell identity, glucose sensing and the control of insulin secretion. Biochem. J..

[49] Naatz A., Yeo C.T., Hogg N., Corbett J.A. (2024). β-Cell-selective regulation of gene expression by nitric oxide. Am. J. Physiol. Regul. Integr. Comp. Physiol..

[50] Badorff C., Fichtlscherer B., Rhoads R.E., Zeiher A.M., Muelsch A., Dimmeler S. (2000). Nitric oxide inhibits dystrophin proteolysis by coxsackieviral protease 2A through S-nitrosylation: a protective mechanism against enteroviral cardiomyopathy. Circulation.

[51] Badorff C., Fichtlscherer B., Muelsch A., Zeiher A.M., Dimmeler S. (2002). Selective delivery of nitric oxide to a cellular target: a pseudosubstrate-coupled dinitrosyl-iron complex inhibits the enteroviral protease 2A. Nitric Oxide.

[52] Colasanti M., Persichini T., Venturini G., Ascenzi P. (1999). S-nitrosylation of viral proteins: molecular bases for antiviral effect of nitric oxide. IUBMB Life.

[53] Tai A.W., Bojjireddy N., Balla T. (2011). A homogeneous and nonisotopic assay for phosphatidylinositol 4-kinases. Anal Biochem..

[54] Yeo C.T., Stancill J.S., Oleson B.J., Schnuck J.K., Stafford J.D., Naatz A. (2021). Regulation of ATR-dependent DNA damage response by nitric oxide. J. Biol. Chem..

[55] Yeo C.T., Kropp E.M., Hansen P.A., Pereckas M., Oleson B.J., Naatz A. (2023). β-cell-selective inhibition of DNA damage response signaling by nitric oxide is associated with an attenuation in glucose uptake. J. Biol. Chem..

[56] Scarim A.L., Heitmeier M.R., Corbett J.A. (1997). Irreversible inhibition of metabolic function and islet destruction after a 36-hour exposure to interleukin-1beta. Endocrinology.

[57] Lacy P.E., Kostianovsky M. (1967). Method for the isolation of intact islets of Langerhans from the rat pancreas. Diabetes.

[58] McDaniel M.L., Colca J.R., Kotagal N., Lacy P.E. (1983). A subcellular fractionation approach for studying insulin release mechanisms and calcium metabolism in islets of Langerhans. Methods Enzymol..

[59] Kelly C.B., Blair L.A., Corbett J.A., Scarim A.L. (2003). Isolation of islets of Langerhans from rodent pancreas. Methods Mol. Med..

[60] Bae Y.S., Eun H.M., Yoon J.W. (1989). Genomic differences between the diabetogenic and nondiabetogenic variants of encephalomyocarditis virus. Virology.

[61] Khan P., Idrees D., Moxley M.A., Corbett J.A., Ahmad F., von Figura G. (2014). Luminol-based chemiluminescent signals: clinical and non-clinical application and future uses. Appl. Biochem. Biotechnol..

