# Supplementary material for: Nitric oxide attenuates PI4P accumulation at the ER membrane to inhibit encephalomyocarditis virus replication selectively in β-cells
Source: J Biol Chem. 2025 Oct 9;301(12):110798. doi: 10.1016/j.jbc.2025.110798 (PMC12639437; doi:10.1016/j.jbc.2025.110798)
Supplement: Sup fig leg [file mmc1.docx]

**Supplemental Figure 1. Uniform display range correction for basal PI4P to allow for PI4P accumulate visualization.** MIN6 cells (600,000 cells/800 µl) were plated on pre-coated optical plates and were cultured or infected with EMCV for 12 hours. Nuclear stain (DAPI, blue) and PI4P (orange) were visualized by immunofluorescence microscopy at 40x magnification. Display ranges were uniformly adjusted across all images to allow for visualization of basal PI4P accumulation in untreated and EMCV-infected cells (top). Display range corrections were made for basal PI4P to allow for optimal visualization of PI4P accumulate formation (bottom).

**Supplemental Table 1. RT-qPCR primer sequences.** “m” indicates species – mus musculus

**Supplemental Table 2. siRNA sequences.**
