# Supplementary material for: Nitric oxide attenuates PI4P accumulation at the ER membrane to inhibit encephalomyocarditis virus replication selectively in β-cells
Source: J Biol Chem. 2025 Oct 9;301(12):110798. doi: 10.1016/j.jbc.2025.110798 (PMC12639437; doi:10.1016/j.jbc.2025.110798)
Supplement: Figure S1 [file mmc2.pdf]

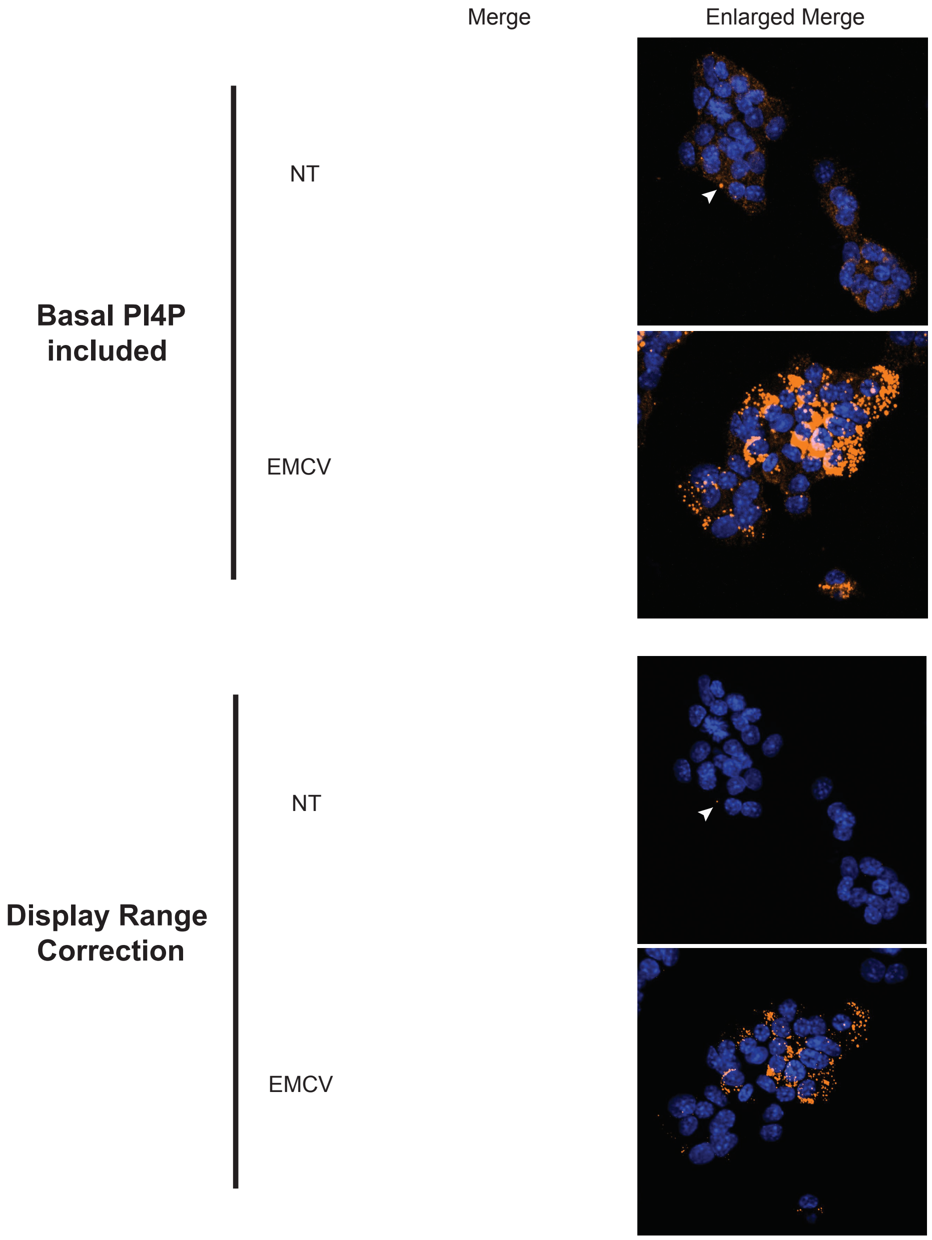

Supplemental Figure 1. Uniform display range correction for basal PI4P to allow for PI4P accumulate visualization.
