# Supplementary material for: Nitric oxide attenuates PI4P accumulation at the ER membrane to inhibit encephalomyocarditis virus replication selectively in β-cells
Source: J Biol Chem. 2025 Oct 9;301(12):110798. doi: 10.1016/j.jbc.2025.110798 (PMC12639437; doi:10.1016/j.jbc.2025.110798)
Supplement: Table S1 [file mmc3.pdf]

Target	Forward Primer Sequence (5’-3’)	Reverse Primer Sequence (5’-3’)
m-GAPDH	GAC ATC AAG AAG GTG GTG AAG C	TCC AGG GTT TCT TAC TCC TTG G
m-EMCV VP1	GGA GTT GAG AAT GCT GAG AG	TTC CAG CAT AAG GAC TTC AG
m-PI4KA	CTG GAG GGG AAA AGA GCC AG	CCT CAT ACC ACT CTT CCG GC
m-PI4KB	ACA ACT AGT GTT CCT GCC CG	AGG GTC TCG TTT GA GGC TG

Supplemental Table 1. qPCR Primers.
