# Supplementary material for: Nitric oxide attenuates PI4P accumulation at the ER membrane to inhibit encephalomyocarditis virus replication selectively in β-cells
Source: J Biol Chem. 2025 Oct 9;301(12):110798. doi: 10.1016/j.jbc.2025.110798 (PMC12639437; doi:10.1016/j.jbc.2025.110798)
Supplement: Table S2 [file mmc4.pdf]

Target:	Abbreviation	Sequence (5’-3’)
Negative Control	siNC	CGU UAA UCG CGU AUA AUA CGC GUA T
PI4KA	siPI4KA	AGG AUA AAG CUA UUC AAA AAG ACA A
PI4KB	siPI4KB	GGC AUG AUU GAA CCA GUA GUC AAC G

Supplemental Table 2. siRNA sequences.
